# Association of Left Atrial Deformation Analysis by Speckle Tracking Echocardiography With Left Atrial Appendage Thrombus in Patients With Primary Valvular Heart Disease

**DOI:** 10.7759/cureus.35151

**Published:** 2023-02-18

**Authors:** Sahar Abdelhamid, Reda Biomy, Hamza Kabil, Mahmoud Raslan, Shaimaa Mostafa

**Affiliations:** 1 Cardiovascular Medicine, King Abdulaziz Specialist Hospital, Sakaka, SAU; 2 Cardiovascular Medicine, Faculty of Medicine, Kafr El Sheikh University, Kafr El Sheikh, EGY; 3 Cardiovascular Medicine, Faculty of Medicine, Damietta University, Damietta, EGY; 4 Cardiovascular Medicine, King Fahd Hospital of the University, Al Khobar, SAU; 5 Cardiovascular Medicine, Faculty of Medicine, Benha University, Benha, EGY

**Keywords:** electrocardiography, valvular heart disease, left atrial appendage thrombus, speckle tracking echocardiography, left atrial deformation

## Abstract

Background: This study aimed to investigate the correlation between the functional parameters of the left atrium (LA) derived from deformation imaging, two-dimensional (2D) speckle tracking echocardiography (STE), and tissue Doppler imaging (TDI) strain and strain rate (SR) and the function of the left atrial appendage (LAA) as measured by transesophageal echocardiography (TEE) in patients with primary valvular heart disease.

Methods: This cross-sectional research included 200 primary valvular heart disease cases, categorized into Group I (n = 74) with thrombus and Group II (n = 126) without thrombus. All patients were subjected to standard 12 lead electrocardiography, transthoracic echocardiography (TTE), strain and SR imaging of the LA by TDI and 2D speckle tracking, and TEE.

Results: At a cut-off value of <10.50%, peak atrial longitudinal strain (PALS) is a predictor of thrombus with an area under the curve (AUC) of 0.975 (95% CI: 0.957-0.993), sensitivity of 94.6%, specificity of 93.7%, positive predictive value (PPV) of 89.7%, negative predictive value (NPV) of 96.7%, and accuracy of 94%. At a cut-off value of <0.295 m/s, LAA emptying velocity is a predictor of thrombus with an AUC of 0.967 (95% CI: 0.944-0.989), sensitivity of 94.6%, specificity of 90.5%, PPV of 85.4%, NPV of 96.6%, and accuracy of 92%. The PALS (<10.50%) and LAA velocity (<0.295 m/s) are significant predictors of thrombus (P = 0.001, β = 2.745, SE = 0.804, OR = 15.56, and 95% CI: 3.219-75.245; and P = 0.002, β = 2.499, SE = 0.799, OR = 12.17, and 95% CI: 2.543-58.201, respectively). Peak systolic strain < 12.55% and SR < 1.065/s are insignificant predictors of thrombus (β = 1.167, SE = 0.996, OR = 3.21, and 95% CI: 0.456-22.631; and β = 1.443, SE = 0.929, OR = 4.23, and 95% CI: 0.685-26.141, respectively).

Conclusions: Among LA deformation parameters derived from TTE, PALS is the best predictor of reduced LAA emptying velocity and LAA thrombus in primary valvular heart disease, regardless of the rhythm.

## Introduction

The left atrium (LA) controls left ventricular filling and cardiac output by acting as a booster pump in late ventricular diastole, as a reservoir for the volume of input from the pulmonary veins throughout ventricular systole and isovolumic relaxation, and as a passive conduit throughout early ventricular diastole and diastasis [1,2]. Loss of LA function has been shown to significantly predict morbidity and mortality in healthy people and patients with various pathologic conditions [3].

A comprehensive investigation of the LA by echocardiography is a method that has been thoroughly validated for assessing the structure and function of the LA. Both components provide crucial information for predicting impending cardiovascular events and risk categorizing existing heart disease cases like heart failure, atrial arrhythmias, valvulopathies, and diastolic dysfunction. Although two-dimensional (2D) techniques for LA assessment constitute the basis of clinical practice, three-dimensional (3D) methods are becoming increasingly popular. A correct assessment of LA volumes (LAVs) has been considerably accelerated by 3D echocardiography [4].

Brain stroke is considered one of the leading causes of morbidity and mortality worldwide [5]. The heart must be evaluated in these patients. The most prevalent reasons for cardiac embolism are decreased ejection fraction (EF), substantial valvular disorder, atrial fibrillation (AF), interatrial septal anomalies (patent foramen ovale, atrial septal defect, and aneurysm), and atherosclerotic plaques from the aorta. Transesophageal echocardiography (TEE) is the best imaging technique for identifying the origins of cardiac embolism [6]. However, invasive measurements of the LA’s function are impossible in most cases [7].

Moreover, the LA’s phased action measures show considerable potential, but a lack of normative information constrains their clinical utility [4]. Measuring the LA’s deformation is a relatively new technology that monitors the LA’s phasic function and enables early diagnosis of subclinical cardiac disease, even in individuals with a typical LA size. Such methods could overcome volumetric evaluation limits, which depend on geometric assumptions and loading conditions [8].

The atrial strain has been investigated in numerous illnesses, such as diabetes, ischemic cardiac disorder, AF, hypertension, and heart failure, in addition to facilitating stroke risk computation and evaluating prognostic consequences [9]. Numerous studies found a correlation between LA’s deformation parameters and left atrial appendage (LAA) function; however, most included cases with AF [10].

This work aimed to evaluate the correlations between the functional parameters of the LA from deformation imaging, 2D speckle tracking echocardiography (STE), and tissue Doppler imaging (TDI) strain and strain rate (SR) and the function of the LAA from TEE findings in patients with primary valvular heart disease.

## Materials and methods

Study design, setting, and date

This cross-sectional research included 200 cases of primary valvular heart disease. The study was conducted in the Cardiology Department, Faculty of Medicine, Benha University Hospital, Egypt, from December 2019 to February 2022.

Ethical concerns

The study obtained approval from the Ethics Committee of the Faculty of Medicine, Benha University, Egypt (REC-FOMBU-13-1-2019). All patients provided written informed consent before participating in the study. Each patient was assigned a unique file with a unique code number. All investigations and findings were solely used for scientific purposes, with strict adherence to patient confidentiality. Unexpected risks that arose during the research were promptly communicated to the participants and the ethical committee.

Eligibility criteria

We included cases aged more than 18 years of both sexes with primary valvular heart disease who were referred to Benha University Hospital for TEE assessment for clinical evidence such as planned cardioversion, routine assessment before the AF ablation procedure, evaluation of intracardiac masses, and evaluation of suitability for valvular repair or transcatheter valvular intervention. We excluded patients with secondary valvular heart disease, patients who refused to undergo TEE, and patients who were contraindicated for TEE.

Procedure

Cases were categorized into two groups based on the presence of thrombus: Group I, which included 74 cases with thrombus, and Group II, which included 126 cases without thrombus. A full history, including smoking, hypertension, diabetes mellitus (DM), and dyslipidemia, was recorded for all cases. Additionally, they had a complete physical examination including body surface area (BSA), first medical contact vital signs, SBP at performance, and heart rate (HR), besides local inspection of the heart, laboratory tests (complete blood count, renal and liver function tests, lipid profile [total cholesterol, low-density lipoproteins, triglycerides, high-density lipoproteins], and HbA1C), standard 12-lead surface electrocardiogram (ECG), transthoracic echocardiography (TTE), evaluation of left ventricular ejection fraction (LVEF) by modified Simpson’s method, strain and SR imaging of the LA by TDI and 2D speckle tracking, and TEE.

Transthoracic echocardiography

A TTE was conducted for each patient. A GE Vingmed Ultrasound AS (Horten, Norway) equipped with a 2D 3.5-MHz transducer (M5S-D), 3D 3.5-MHz transducer (4C-D), offline speckle tracking analysis software, and background processing workstation (EchoPAC BT 11.1.0, GE Medical System, Horten, Norway) was used to acquire echocardiographic data. Standard parasternal and apical imaging pictures were taken with the patient on the left side. Transthoracic echocardiography was performed to detect valvular pathology, assess the severity of valvular disease, assess left ventricular systolic function, and evaluate LA deformation by different parameters, including 2D speckle tracking imaging.

Evaluation of left ventricular ejection fraction by modified Simpson’s technique

Volumetric measurements were performed by identifying the interaction between the compacted myocardium and the left ventricle (LV) cavity in the apical four- and two-chamber views [11]. The EF was determined by the formula: EF = (End-diastolic volume - End-systolic volume)/End-diastolic volume.

Assessment of left atrial size

Left atrial volume was estimated using the biplane area-length (AL) technique utilizing four- and two-chamber views (4C-2C). The maximum LA volume was measured during end-ventricular systole, right before the mitral valve opening, from the lateral aspect of the mitral annulus to the septal aspect, not including the region between the leaflets and annulus, and after the LA endocardium, not including the pulmonary veins and LAA. The minimum LA volume was obtained at the end-diastole on the frame, just before mitral valve closure. The length of the LA was measured as a perpendicular line determined from the middle of the plane extending from the mitral annulus to the superior aspect of the LA [11]. The volume was estimated based on the formula: (0.85 * Four-chamber area * Two-chamber area)/(Longest LA length). The LA volume index (LAVI) was determined by dividing the volumes by the BSA.

Assessment of left atrial distensibility

The left atrial distensibility was calculated from the following formula: (Vmax) - (Vmin) × 100/(Vmin), where (Vmax) is the maximal atrial volume measured at end-systole (T wave on ECG) and (Vmin) is the minimum atrial volume measured at end-diastole (R wave on ECG).

Strain and strain rate imaging of left atrium by tissue Doppler imaging and two-dimensional speckle tracking

Tissue Doppler LA strain and SR were calculated offline from the color TDI of the atria shown in the apical four- and two-chamber views at high frame rates (>100 fps). A limited sample amount (10 mm × 2 mm) was chosen because of the thin atrial walls. To minimize artifactual information, the volume of the sample was put in the center of the sector and realigned so that the direction of movement interviewed was a possible close parallel to the direction of the insonating beam to prevent interference from mitral annular motion. In the apical four-chamber view, the septal and lateral walls are visible; in the apical two-chamber view, the inferior and anterior walls are visible.

Strain and strain rate by two-dimensional speckle tracking

The LA deformation was articulated in three phases in each cardiac cycle, the reservoir strain (εR), the conduit strain (εCD = left atrial conduit strain), and the contractile strain (εCT = left atrial contractile strain). All cases were attached to the ECG throughout the assessment and kept in the left lateral decubitus position. Standard 2D grayscale echocardiography was used to capture pictures of the apical four- and two-chamber views through a breath-hold with a steady ECG record for speckle tracking investigation. The mean of three successive cardiac cycles was observed. Point-and-click detection and labeling of reference landmarks (annulus and roof) in the four- and two-chamber views were displayed at 60 and 80 frames per second, respectively. The LA endocardial surface and adjusted region of interest width were tracked. Following manual correction of the breadth and geometry of the region of interest, the software segmented the region of interest into six sections, and each segment’s tracking quality was automatically rated as acceptable or unacceptable, with the option of manual modification. Following user approval, segmental longitudinal strain curves were produced. The PALS, measured at the end of the reservoir stage, and peak atrial contraction strain, as estimated before the beginning of the active atrial contractile stage, are determined using average values detected in all LA segments independently, averaging four- and two-chamber observations. The reference ranges for PALS are shown in Table 1 [12].

**Table 1:** Reference values for peak atrial longitudinal strain LA: Left atrial; CI: Confidence interval.

LA strain component	Number of studies	Mean	95% CI	Cochrane Q	I2	t2
Reservoir	40	39.4	38.0–40.8	1,653 (P < .001)	97.6	20.0
Conduit	14	23.0	20.7–25.2	420 (P < .001)	96.9	17.9
Contractile	18	17.4	16.0–19.0	631 (P < .001)	97.3	9.7

Transesophageal echocardiography

A TEE was conducted for each patient to assess the LAA flow velocity and search for the presence of LAA thrombus. After obtaining the results of 2D LA speckle tracking and TEE, the findings of 2D LA speckle tracking peak longitudinal strain and TEE were compared in terms of predicting LAA flow velocity and the presence of LAA thrombus. In normal hearts during sinus rhythm, typical filling velocities range from 46 to 60 cm/s [13].

Statistical analysis

The Windows version of the Statistical Package for Social Sciences (SPSS) version 26 (IBM Corp., Armonk, NY) was utilized to examine the data. The data’s normality was first evaluated using a Kolmogorov-Smirnov test on a single sample. Number and percentage descriptions were provided for qualitative data. Using the Chi-square test, the association between categorical data was investigated. Continuous variables with normally distributed data were given as mean and SD (standard deviation), and the student's t-test was used to compare the two groups. Using Pearson correlation, continuous data were correlated. A two-tailed P-value of 0.05 was deemed statistically significant.

## Results

Demographic data, risk factors, and anticoagulants among the studied group are shown in Table 2.

**Table 2:** Demographic data, risk factors, and anticoagulants among the studied group Data are represented as mean ± SD or frequency (%). HTN: Hypertension; DM: Diabetes mellitus; BSA: Body surface area.

Demographic data		The study group (n = 200)
Age (years)		44.16 ± 13.59
Gender	Male	102 (51.0%)
	Female	98 (49.0%)
BSA (m^2^)		1.72 ± 0.12
HTN		48 (24.0%)
DM		38 (19.0%)
Smokers		76 (38.0%)
Anticoagulant		36 (18.0%)

Table 3 shows ECG, LA diameter, LA distension, LAV, LA strain (TDI), LA strain (speckle), and TEE among the studied group.

**Table 3:** The ECG, LA diameter, LA distension, LAV, LA strain (TDI), LA strain (speckle), and TEE among the studied group Data are represented as mean ± SD or frequency (%). ECG: Electrocardiogram; AF: Atrial fibrillation; LVEF: Left ventricular ejection fraction; LA: Left atrium; LAV: Left atrial volume; LAVI: Left atrial volume index; SR: Strain rate; TDI: Tissue Doppler imaging; εR: Reservoir strain; εCD: Conduit strain; εCT: Contractile strain; PALS: Peak atrial longitudinal strain; TEE: Transesophageal echocardiogram; LAA: Left atrial appendage.

ECG and LVEF	The study group (n = 200)
ECG	
AF	48 (24.0%)
Sinus rhythm	152 (76.0%)
LVEF (%)	55.87 ± 9.71
LA diameter (cm)	4.76 ± 0.95
LA distension (%)	29.52 ± 12.99
LAV	
MAX (mL)	87.98 ± 33.98
MIN (mL)	64.85 ± 32.93
LAVI (mL/m^2^)	51.44 ± 20.68
LA deformation	
LA strain (TDI)	
Peak sys strain (%)	11.82 ± 3.54
SR (/sec)	1.11 ± 0.36
LA strain (speckle)	
εR (%)	16.13 ± 9.06
εCD (%)	-9.79 ± 3.47
εCT (%)	-8.32 ± 4.37
PALS (%)	16.13 ± 9.06
TEE	
LAA velocity (m/s)	0.45 ± 0.28
Thrombus	74 (37.0%)

There is a significant positive correlation between LAA velocity and LVEF (r = 0.160, P = 0.024), LA distension (r = 0.748, P ≤ 0.001), peak systolic strain (r = 0.615, P ≤ 0.001), SR (r = 0.623, P ≤ 0.001), εR (r = 0.965, P ≤ 0.001), and PALS (r = 0.965, P ≤ 0.001). There is a significant negative association between LAA velocity and LA diameter (r = -0.860, P ≤ 0.001), LAV MAX (r = -0.904, P ≤ 0.001), LAV MIN (r = -0.894, P ≤ 0.001), LAVI (r = -0.888, P ≤ 0.001), εCD (r = -0.884, P ≤ 0.001), and εCT (r = -0.957, P ≤ 0.001). There is no significant correlation between LAA velocity and BSA (Table 4).

**Table 4:** Association between the left atrial appendage velocity and other variables BSA: Body surface area; LVEF: Left ventricular ejection fraction; LA: Left atrium; LAV: Left atrial volume; LAVI: Left atrial volume index; SR: Strain rate; εR: Reservoir strain; εCD: Conduit strain; εCT: Contractile strain; PALS: Peak atrial longitudinal strain. * Significant as P-value ≤ 0.05.

Variables	Left atrial appendage velocity	
	r	P-value
BSA (m^2^)	0.095	0.181
LVEF (%)	0.160	0.024*
LA diameter (cm)	-0.860	≤0.001*
LA distension (%)	0.748	≤0.001*
LAV MAX (mL)	-0.904	≤0.001*
LAV MIN (mL)	-0.894	≤0.001*
LAVI (mL/m^2^)	-0.888	≤0.001*
peak sys strain (%)	0.615	≤0.001*
SR (/sec)	0.623	≤0.001*
εR (%)	0.965	≤0.001*
εCD (%)	-0.884	≤0.001*
εCT (%)	-0.957	≤0.001*
PALS (%)	0.965	≤0.001*

Age, LAV (MAX, MIN, and LAVI), LA diameter, mitral stenosis (MS), and AF are significantly higher in the thrombus group than in the non-thrombus group (P ≤ 0.001). The number of males, BSA, LA strain by TDI (peak systolic strain and SR), international normalized ratio (INR), LA distension, mitral regurgitation (MR), and sinus rhythm are significantly lower in the thrombus group than in the non-thrombus group (P ≤ 0.001). There is no significant difference in hypertension, DM, smoking, aortic stenosis, and aortic regurgitation among the study group. LVEF was significantly lower in the thrombus group than in the non-thrombus group (P = 0.001). None of the patients who had sinus rhythm received anticoagulants in either group. Anticoagulant administration among patients with AF is not significantly different between the thrombus and non-thrombus groups (Table 5).

**Table 5:** Sociodemographic data, cardiac data, and cardiac rhythm in the studied groups HTN: Hypertension; DM: Diabetes mellitus; BSA: Body surface area; ECG: Electrocardiogram; AF: Atrial fibrillation; MS: Mitral stenosis; MR: Mitral regurgitation; AS: Aortic stenosis; AR: Aortic regurgitation; LVEF: Left ventricular ejection fraction; LA: Left atrium; LAV: Left atrial volume; LAVI: Left atrial volume index; SR: Strain rate; TDI: Tissue Doppler imaging; εR: Reservoir strain; εCD: Conduit strain; εCT: Contractile strain; PALS: Peak atrial longitudinal strain; INR: International normalized ratio. * Significant as P-value ≤ 0.05.

Sociodemographic data		Thrombus		p-value
		Group I (Yes) (n = 74)	Group II (No) (n = 126)	
Age (years)		46.72 ± 11.656	42.65 ± 14.44	≤0.001*
Gender	Male	22 (29.7%)	80 (63.5%)	≤0.001*
	Female	52 (70.3%)	46 (36.5%)	
BSA (m^2^)		1.68 ± 0.11	1.74 ± 0.11	≤0.001*
HTN		18 (24.3%)	30 (23.8%)	0.934
DM		18 (24.3%)	20 (15.9%)	0.141
Smoker		26 (35.1%)	50 (39.7%)	0.522
Cardiac data				
ECG	AF	40 (54.1%)	8 (6.3%)	≤0.001*
	Sinus	34 (45.9%)	118 (93.7%)	
LVEF (%)		53.05 ± 10.21	57.53 ± 9.04	0.001*
Valve pathology				
MS		62 (83.8%)	32 (25.4%)	≤0.001*
MR		4 (45.9%)	56 (44.4%)	0.028*
AS		4 (5.4%)	20 (15.9%)	0.837
AR		40 (54.1%)	52 (41.3%)	0.08
LA diameter (cm)		5.62 ± 0.58	4.26 ± 0.73	≤0.001*
LA distension (%)		19.64 ± 6.18	35.32 ± 12.44	≤0.001*
LAV				
MAX (mL)		120.10 ± 16.53	69.12 ± 26.64	≤0.001*
MIN (mL)		96.47 ± 15.80	46.28 ± 25.32	≤0.001*
LAVI (mL/m^2^)		71.46 ± 10.64	39.68 ± 15.43	≤0.001*
LA strain (TDI)				
Peak sys strain (%)		8.88 ± 2.30	13.54 ± 2.98	≤0.001*
SR (/sec)		0.87 ± 0.17	1.25 ± 0.37	≤0.001*
LA strain (speckle)				
εR (%)		7.59 ± 1.47	21.14 ± 7.82	≤0.001*
εCD (%)		-6.56 ± 1.66	-11.68 ± 2.81	≤0.001*
εCT (%)		-2.41 ± 1.20	-10.03 ± 3.33	≤0.001*
PALS (%)		7.59 ± 1.47	21.14 ± 7.82	≤0.001*
INR		2.03 ± 0.25	2.51 ± 0.07	≤0.001*
Sinus				
Anticoagulant		0/34 (0%)	0/118 (0%)	-
No anticoagulant		34/34 (100%)	118/118 (100%)	
AF				
Anticoagulant		28/40 (70%)	8/8 (100%)	0.174
No anticoagulant		12/40 (30%)	0/8 (0%)	

**Figure 1:**
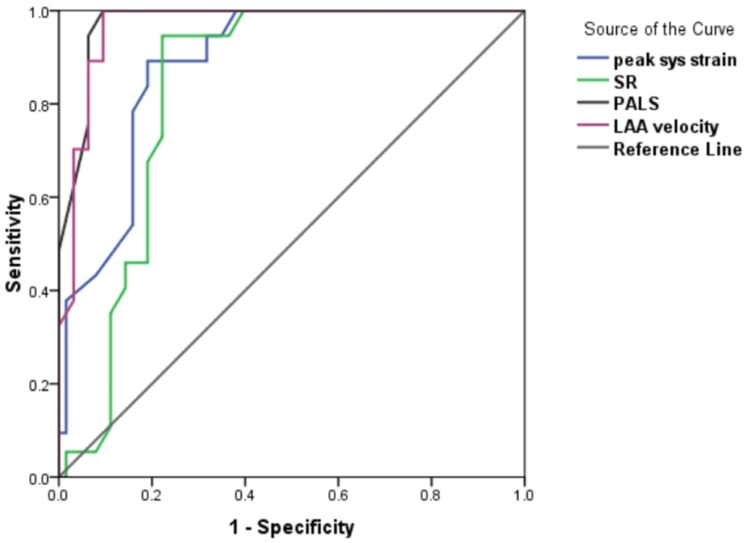
Receiver operating characteristic curve for the prediction of thrombus by peak sys strain, SR, PALS, and LAA velocity SR: Strain rate; PALS: Peak atrial longitudinal strain; LAA: Left atrial appendage.

At a cut-off value of <12.55%, peak SS is a predictor of thrombus with an area under the curve (AUC) of 0.885 (95% CI: 0.840-0.929), 94.6%, sensitivity, 68.3% specificity, 63.6% positive predictive value (PPV), 95.6% negative predictive value (NPV), and 78% accuracy. At a cut-off value of <1.065/s, SR is a predictor of thrombus with an AUC of 0.831 (95% CI: 0.773-0.890), 94.6% sensitivity, 77.8% specificity, 71.4% PPV, 96.1% NPV, and 84% accuracy. At a cut-off value of <10.50%, the PALS is a predictor of thrombus with an AUC of 0.975 (95% CI: 0.957-0.993), 94.6% sensitivity, 93.7% specificity, 89.7% PPV, 96.7% NPV, and 94% accuracy. At a cut-off value of <0.295 m/s, LAA emptying velocity is a predictor of thrombus with an AUC of 0.967 (95% CI: 0.944-0.989), 94.6% sensitivity, 90.5% specificity, 85.4% PPV, 96.6% NPV, and 92% accuracy (Figure 1).

In multiple logistic regression analysis, the PALS (<10.50%) and LAA velocity (<0.295 m/s) are significant predictors of thrombus: (P = 0.001, β = 2.745, SE = 0.804, OR = 15.56, and 95% CI: 3.219-75.245) and (P = 0.002, β = 2.499, SE = 0.799, OR = 12.17, and 95% CI: 2.543-58.201), respectively. Peak systolic strain (<12.55%) and SR (<1.065/s) are insignificant predictors of thrombus: (β = 1.167, SE = 0.996, OR = 3.21, and 95% CI: 0.456-22.631) and (β = 1.443, SE = 0.929, OR = 4.23, and 95% CI: 0.685-26.141), respectively. In multiple logistic regression analysis, the PALS (<10.50%) is a significant predictor of LAA velocity < 0.2 m/s: (P < 0.001, β = 4.775, SE = 0.740, OR = 118, and 95% CI: 27-505). Peak systolic strain (<12.55%) and SR (<1.065 /s) are insignificant predictors of LAA velocity < 0.2 m/s: (β = 0.153, SE = 0.814, OR = 1.165, and 95% CI: 0.221-6.13) and (β = 1.146, SE = 0.814, OR = 3.146, and 95% CI: 0.638-15.52), respectively (Table 6).

**Table 6:** Multiple logistic regression analysis for the predictors of thrombus and left atrial appendage velocity < 0.2 m/s OR: Odds ratio; CI: Confidence interval, cut-off point based on receiver operating characteristic (ROC) curve; SE: Standard error; SS: Sys strain; SR: Strain rate; PALS: Peak atrial longitudinal strain; LAA: Left atrial appendage. * Significant as P-value ≤ 0.05.

	β	SE	P-value	OR	95% CI	
Predictors of thrombus						
Peak sys strain (<12.55)	1.167	0.996	0.241	3.21	0.456	22.631
SR (<1.065)	1.443	0.929	0.120	4.23	0.685	26.141
PALS (<10.50)	2.745	0.804	0.001*	15.56	3.219	75.245
LAA velocity (<0.295)	2.499	0.799	0.002*	12.17	2.543	58.201
Predictors of LAA velocity < 0.2 m/s						
Peak sys strain (<12.55)	0.153	0.847	0.857	1.165	0.221	6.13
SR (<1.065)	1.146	0.814	0.159	3.146	0.638	15.52
PALS (<10.50)	4.775	0.740	≤0.001*	118	27	505

**Figure 2:**
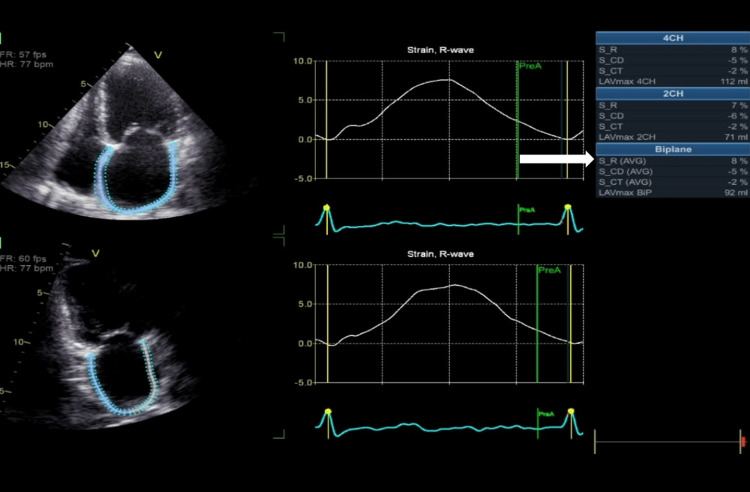
Measurement of left atrial strain components using the zero-strain reference at end-diastole. Note the reduced peak atrial longitudinal strain of 8% (arrow).

**Figure 3:**
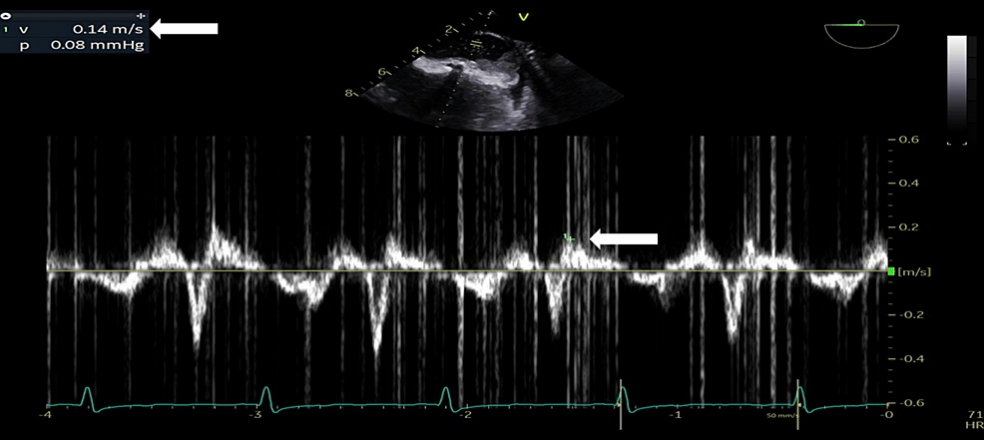
Transesophageal echocardiography: pulsed wave Doppler at the opening of the left atrial appendage. Note the low left atrial appendage velocity of 14 cm/s (arrow).

**Figure 4:**
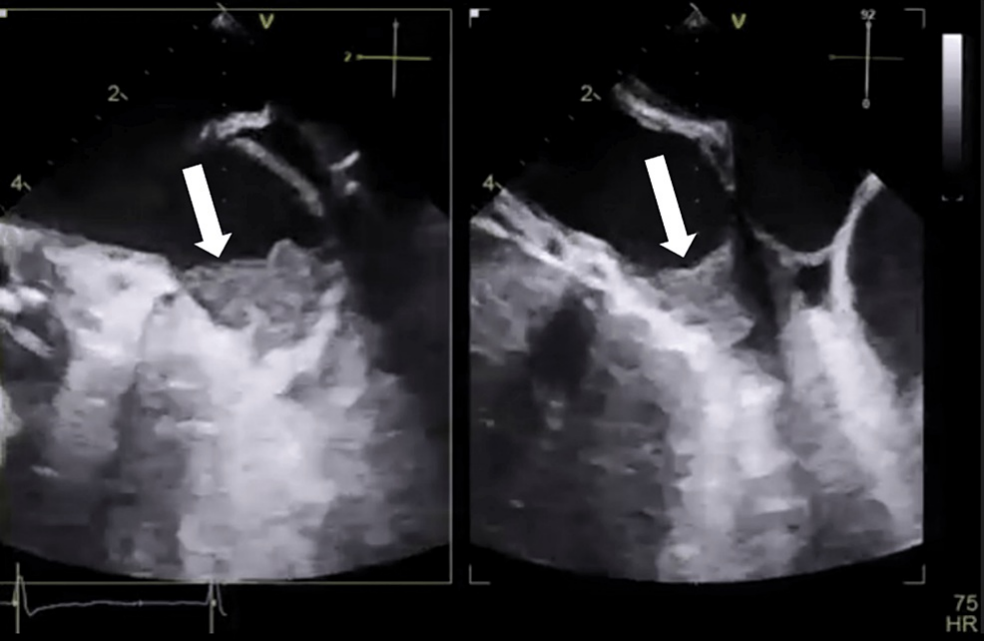
Transesophageal echocardiography mid-esophageal left atrial appendage view. Note the large thrombus occupying most of the left atrial appendage (arrow).

A 31-year-old woman with a medical history of rheumatic heart disease arrived at the emergency department with an acute stroke. This woman had no known history of hypertension or diabetes, and her TTE shows normal left ventricular systolic function, EF of 66%, severe bi-atrial dilatation, rheumatic appearing mitral valve with thickening and calcified leaflet tips, moderate to severe MR, severe MS (the estimated mitral valve area was 0.9 cm^2^ by 2D planimetry, and the mean gradient was 13 mmHg), rheumatic appearing aortic valve with severe aortic regurgitation, rheumatic appearing tricuspid valve with severe tricuspid regurgitation, a severely dilated right ventricle, a normal systolic function, and a severely increased pulmonary pressure (right ventricular systolic pressure was 80 mmHg). LA speckle tracking showed that LA strain (in the reservoir, conduit, and contractile phases) is markedly reduced (PALS: 8%) (Figures 2-4).

**Figure 5:**
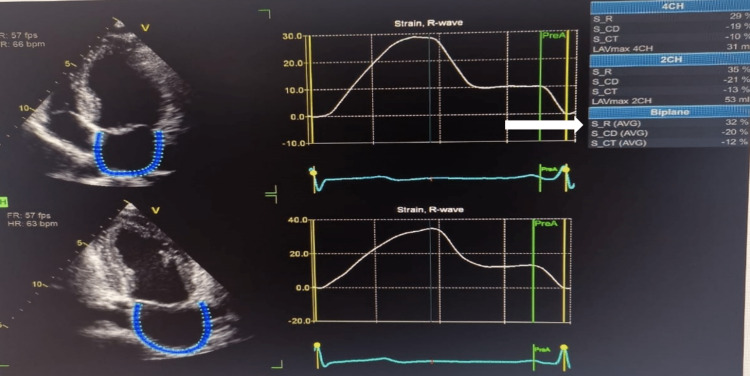
Measurement of left atrial strain components using the zero-strain reference at end-diastole. Note the peak atrial longitudinal strain of 32% (arrow).

**Figure 6:**
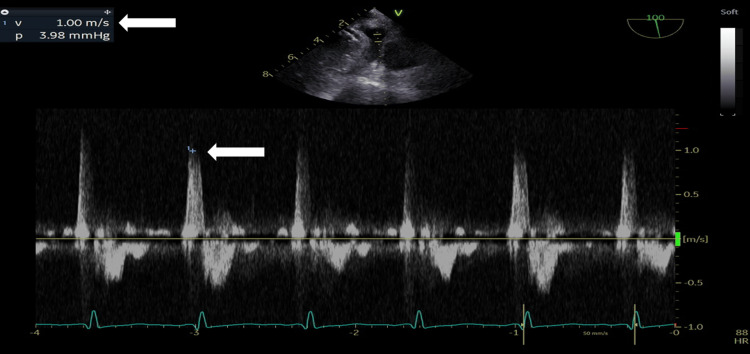
Transesophageal echocardiography mid-esophageal left atrial appendage view at 100˚, pulsed wave Doppler at the opening of the left atrial appendage. Note the left atrial appendage velocity of 1.00 m/s (arrow).

**Figure 7:**
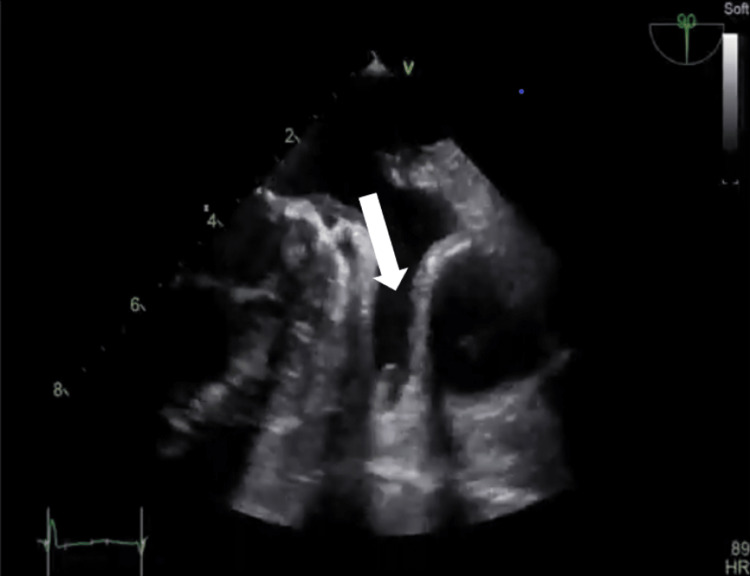
Transesophageal echocardiography focusing on two-dimensional images of the clear left atrial appendage. Note the pectinate muscle that might be mistaken as a thrombus, ruled out using the contrast (arrow).

A 25-year-old man who was diagnosed with a bicuspid aortic valve and moderate aortic regurgitation when he was 19 years old came to our cardiology clinic as a regular follow-up, and his TTE shows a mildly dilated LV, no hypertrophy, a normal global LV systolic function, EF of 58%, a bicuspid aortic valve, fused right coronary cusp and left coronary cusp with raphe, severe aortic regurgitation, an eccentric posteriorly directed jet hitting anterior mitral leaflet, vena contracta width of 0.6 cm, an effective regurgitant orifice area of 0.4 cm^2^, regurgitant volume of 62 mL, regurgitant fraction of 53%, diastolic flow reversal in descending thoracic aorta, and dilated proximal ascending aorta measuring 4.2 cm. LA speckle tracking showed that LA strain (in the reservoir, conduit, and contractile phases) was mildly reduced (PALS: 32%) (Figures 5-7).

## Discussion

Cardioembolic stroke is a crucial clinical concern since it is the leading reason for mortality in individuals with an acute ischemic stroke. The LAA thrombus is a key cause of thromboembolism in stroke cases with AF [10].

In the current study, the BSA was found to be significantly lower in the thrombus group than in the non-thrombus group (P ≤ 0.001). Kupczynska et al. [14] has reported similar findings. In contrast, Kurzawski et al. [15] screened about 80 patients with normal LVEF who experienced TEE (40 cases with SR and 40 cases with AF on oral anticoagulants involving LAA thrombus). The findings indicated no significant difference between the thrombus and non-thrombus groups regarding the BSA (P = 0.589). The contradiction between both studies can be justified by having normal LVEF and a small sample size compared to our study.

We found no significant differences concerning hypertension, DM, and smoking between the study groups. Our findings agree with those of Kupczynska et al. [14] who emphasized the absence of significant differences as hypertension, DM, and smoking among the study group (P > 0.005).

In the current study, the anticoagulant administration among cases with AF was not significantly different in the thrombus group compared to the non-thrombus group. Consistent with our research, Kupczynska et al. [14] highlighted that anticoagulant administration among patients with AF was not significantly different among the thrombus and non-thrombus groups.

We found that the LVEF significantly decreased while LAV (LAVI) substantially increased in the thrombus group compared to the non-thrombus group (P = 0.001). This agrees with the research conducted by Wang et al. [16].

The present study found that the LA dimension increased significantly in the thrombus group compared to the non-thrombus group (P ≤ 0.001). In agreement with our results, Natarajan et al. [17] studied 120 cases of rheumatic heart disease (dominant lesion being MS and MR) and cases with cerebrovascular stroke (ischemic or hemorrhagic) with documented evidence (CT or MR imaging). The outcomes showed that LA dimensions increased significantly in the thrombus group compared to the non-thrombus group.

Regarding the valve pathology of the studied groups, MR showed a significantly more reduction in the thrombus group than in the non-thrombus (P ≤ 0.001). In line with our results, Sonaglioni et al. [18] involved all consecutive cases with non-valvular AF. In Group I, a left atrial appendage thrombus (LAAT) was found in 25 cases (21.7%); in Group II, 90 cases without a LAAT (78.3%) were categorized as controls. The results showed that MR decreased significantly in the thrombus group compared to the non-thrombus group (P = 0.01).

In this study, it was found that LA strain by TDI (SR) was significantly lower in the thrombus group than in the non-thrombus group (P ≤ 0.001). Similarly, Sonaglioni et al. [18] highlighted that LA strain by TDI (SR) was significantly lower in the thrombus group than in the non-thrombus group. The results revealed that the PALS significantly decreased in the thrombus group than in the non-thrombus (P = 0.001). Mostafa et al. [10] noticed that the PALS was significantly lower in the thrombus group than in the non-thrombus group (P < 0.001), which is consistent with our findings. Moreover, INR was significantly lower in the thrombus group than in the non-thrombus group (P-value ≤ 0.001). Correspondingly, Zhu et al. [19] highlighted that INR is significantly lower in the thrombus group than in the non-thrombus group.

In the present study, it was found that at a cut-off value of <12.55%, peak SS was a predictor of thrombus with an AUC of 0.885 (95% CI: 0.840-0.929), 94.6% sensitivity, 68.3% specificity, 63.6% PPV, 95.6% NPV, and 78% accuracy. Moreover, Sonaglioni et al. [18] highlighted that receiver operating characteristic (ROC) curve analysis showed that a left atrial reservoir strain cut-off value of ≤9.3% had 98.9% sensitivity and 100% specificity for the detection of LAAT at TEE (AUC: 0.98, P < 0.001).

According to our results, at a cut-off value of <10.50%, the PALS was a predictor of thrombus with an AUC of 0.975 (95% CI: 0.957-0.993), 94.6% sensitivity, 93.7% specificity, 89.7% PPV, 96.7% NPV, and 94% accuracy. Furthermore, at a cut-off value of <0.295 m/s, LAA emptying velocity was a predictor of thrombus with an AUC of 0.967 (95% CI: 0.944-0.989), 94.6% sensitivity, 90.5% specificity, 85.4% PPV, 96.6% NPV, and 92% accuracy. Following our findings, Mostafa et al. [10] highlighted that a 2D PALS cut-off value of <31% had diagnostic accuracy with a sensitivity of 88.2% and a specificity of 77.9% in predicting LAA thrombus or shadow in patients with non-valvular AF (AUC: 0.833, 95% CI: 0.779-0.887, P < 0.001). A 2D PALS rate cut-off value of <1.16 had diagnostic accuracy with a sensitivity of 87.3% and a specificity of 75.7% in predicting LAA thrombus or shadow (AUC: 0.811, 95% CI: 0.754-0.867, P < 0.001). Besides, among all LA deformation parameters derived from TTE, in multiple logistic regression analysis, the PALS was the best predictor of decreased LAA emptying velocity (<0.2 m/s) and LAA thrombus in cases with a primary valvular heart disorder. This finding is similar to that of Cameli et al. [20], who showed that global PALS was the strongest predictor of LAA thrombus or impaired LAA emptying among all measures determined from TTE in cases with persistent non-valvular AF receiving TEE before electrical cardioversion or ablation treatments.

This study has some limitations, such as being a single-center research with a small sample size. In addition, there was no analysis of STE-derived parameters regarding the LAA segment and some patients receiving low-dosage anticoagulation treatment. However, echo parameters are well-established indicators of LAA dysfunction despite this anticoagulation treatment. Moreover, cases with both sinus rhythm and AF were involved in the study, and cases with preserved and depressed systolic function were included in the study.

## Conclusions

There was a significant positive correlation between LAA velocity and LVEF, LA strain by TDI (peak systolic strain and SR), and PALS. Furthermore, MS, AF, LAV (MAX, MIN, and LAVI), and LA diameter were significantly higher, but LVEF, INR, and LA strain by TDI (peak systolic strain and SR) were significantly lower in the thrombus group compared to the non-thrombus group. Moreover, the peak SS, PALS, and LAA may be reliable markers for thrombus formation. Among all LA deformation parameters derived from TTE, PALS was the best predictor of reduced LAA emptying velocity and LAA thrombus in patients with primary valvular heart disease, regardless of the rhythm (sinus or AF rhythm).
